# HDACs and the senescent phenotype of WI-38 cells

**DOI:** 10.1186/1471-2121-6-37

**Published:** 2005-10-26

**Authors:** Robert F Place, Emily J Noonan, Charles Giardina

**Affiliations:** 1 Department of Molecular and Cell Biology, University of Connecticut, Storrs, Connecticut 06269, USA

## Abstract

**Background:**

Normal cells possess a limited proliferative life span after which they enter a state of irreversible growth arrest. This process, known as replicative senescence, is accompanied by changes in gene expression that give rise to a variety of senescence-associated phenotypes. It has been suggested that these gene expression changes result in part from alterations in the histone acetylation machinery. Here we examine the influence of HDAC inhibitors on the expression of senescent markers in pre- and post-senescent WI-38 cells.

**Results:**

Pre- and post-senescent WI-38 cells were treated with the HDAC inhibitors butyrate or trichostatin A (TSA). Following HDAC inhibitor treatment, pre-senescent cells increased p21^WAF1 ^and β-galactosidase expression, assumed a flattened senescence-associated morphology, and maintained a lower level of proteasome activity. These alterations also occurred during normal replicative senescence of WI-38 cells, but were not accentuated further by HDAC inhibitors. We also found that HDAC1 levels decline during normal replicative senescence.

**Conclusion:**

Our findings indicate that HDACs impact numerous phenotypic changes associated with cellular senescence. Reduced HDAC1 expression levels in senescent cells may be an important event in mediating the transition to a senescent phenotype.

## Background

Normal somatic cells possess a limited proliferative life span after which they enter a state of irreversible growth arrest. This process, known as replicative senescence, can be signaled by shortened telomeres that result from repeated rounds of DNA replication in the absence of telomerase expression. Once the telomeres erode to an average size of 4–6 kilobases, senescence is triggered and cells stop dividing [1,2]. Replicative senescence plays an important role in maintaining the structural integrity of tissues by limiting the excessive clonal expansion of cells [3,4]. However, the accumulation of senescent cells is also believed to contribute to the age-related decline in tissue function [5]. Replicative senescence can therefore be viewed as both a mechanism of tumor suppression and a contributor in pathologies associated with age. The role of replicative senescence in tumorigenesis is highlighted by the fact that the most common mutations in human cancers occur in genes encoding p53 and members of the pRB pathway, which are the critical effectors of replicative senescence [4,6,7].

A number of fundamental metabolic and biochemical changes occur as a cell enters senescence and begins to age. Numerous studies have reported dramatic changes in protein turnover. The proteasome, the primary non-lysosomal protease responsible for degrading intracellular proteins including misfolded, oxidized and ubiquitinated proteins, has been reported to decline in function with age [8-13]. Several reports have indicated that the expression of certain proteasome subunits drops after cells enter replicative senescence [14-17]. In addition, proteasome inhibition, or "clogging", has been observed as aging cells accumulate damaged proteins [12,13,18]. The resulting drop in protein turnover may contribute to the accumulation of protein deposits, such as lipofuscin, which can further compromise cell function [19]. In addition, the drop in proteasome activity is likely to alter the activity of numerous cellular signal transduction pathways that involve the proteasome.

Replicative senescence is accompanied by many changes in gene expression that contribute to the senescence-associated phenotypes. Of particular importance are the cell cycle inhibitors p16^INK4a ^and p21^WAF1^, which are induced upon replicative senescence to halt cell proliferation [20,21]. Interestingly, many genes involved in the regulation of cellular growth arrest and differentiation are regulated by histone acetylation. For example, in proliferating fibroblasts, the stable association of HDAC1 with the Sp1/Sp3 transcription factors bound to the p21^WAF1 ^promoter suppresses p21^WAF1 ^expression. Upon senescence, HDAC1 is displaced from to the p21^WAF1 ^promoter, due in part to the actions of p53 [22].

HDAC inhibitors have long been known to induce differentiation, growth arrest, and apoptosis in cancer cells [23-25]. The aberrant utilization of HDACs is believed to be a contributing factor in carcinogenesis. However, only recently have HDAC inhibitors been shown to induce premature senescence in normal human fibroblasts [26,27]. HDACs may therefore play a critical role in modulating cell physiology during the aging process, as well as contribute to the cellular changes associated with transformation. Here we examine the interplay between cellular HDAC activity and a number of phenotypic changes that accompany cell senescence. We find that replicative senescence is accompanied by a drop in cellular HDAC1 expression, the activation of the cell cycle inhibitory protein p21^WAF1^, and a reduction in cellular proteasome activity and subunit expression. The critical role of HDACs in regulating these events is supported by the finding that HDAC inhibitors selectively trigger these changes in pre-senescent, but not post-senescent cells. Our findings indicate that a drop in HDAC expression may be a critical event in mediating the transition from a proliferating to a senescent phenotype.

## Results

### HDAC inhibitors induce a senescence-like phenotype in proliferating WI-38 cells

**Figure 1:**
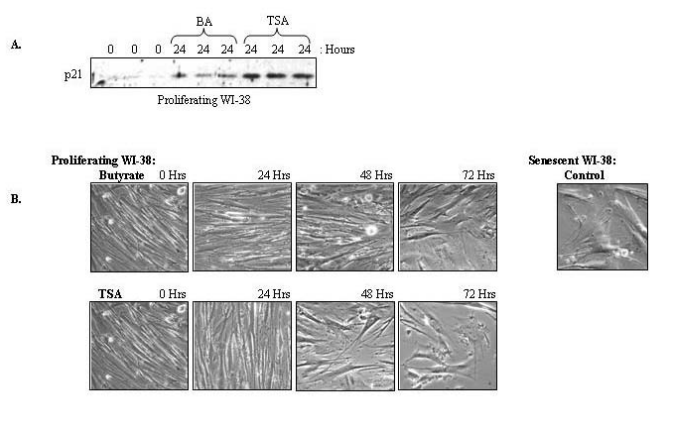
**HDAC inhibition induces markers of senescence**. **(A) **Butyrate and TSA induce the expression of the senescence-associated cell cycle inhibitor protein p21^WAF1^. Proliferating WI-38 cells were treated with butyrate (BA) or TSA for 24 hours. Cytosolic extracts were prepared and equivalent protein levels were analyzed by immunoblot using an anti-p21^WAF1 ^antibody. Untreated samples (0 hours) consisted of cytosolic extracts prepared from naïve cells. Results are shown in triplicate. **(B) **Butyrate and TSA rapidly induce senescent-like morphologies in young WI-38 cells. Proliferating WI-38 cells were treated with butyrate or TSA for 0, 24, 48, and 72 hours, as indicated. Phase contrast images of cell morphology were taken at 100 × magnification. An image of WI-38 cells propagated to replicative senescence is also shown.

HDAC inhibitors can induce growth arrest in many cell types, and have recently been reported to induce a senescence-like state in normal human fibroblasts [26,27]. Therefore, we sought to determine if the HDAC inhibitors butyrate and TSA could induce premature senescence in proliferating WI-38 cells. One molecular marker of senescence in normal human fibroblasts is p21^WAF1 ^expression [28]. As shown in Figure 1A, treatment with butyrate or TSA for 24 hours induced the expression of p21^WAF1 ^in proliferating WI-38 cells. Distinct morphological changes also occurred when WI-38 cells enter replicative senescence. Senescent cells became larger and assumed irregular shapes, while proliferating WI-38 cells formed long and striated parallel arrays (Figure 1B). As shown in Figure 1B, treatment of young WI-38 cells with HDAC inhibitors butyrate or TSA caused cells to rapidly acquire a senescent-like morphology.

**Figure 2:**
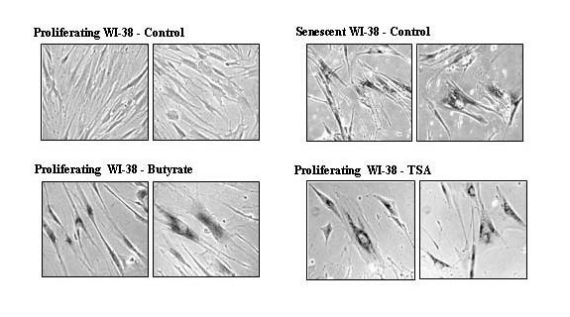
**HDAC inhibition induces the senescence-associated-β-glacatosidase (SA-β-gal) activity in proliferating WI-38 cells**. Proliferating WI-38 cells were propagated for 14 days in 0.5 mM butyrate (Proliferating WI-38; Butyrate) or 9 days in 0.5 μM TSA (Proliferating WI-38; TSA) and stained for SA-β-gal activity. The level of SA-β-gal staining was also determined in untreated young WI-38 cells propagated in parallel (Proliferating WI-38; Control), as well as WI-38 cells propagated to replicative senescence (Senescent WI-38; Control). Phase contrast images are shown in duplicate at 100 × magnification.

Another biomarker for replicative senescence is senescence-associated-β-galactosidase (SA-β-gal) activity [29]. Young WI-38 cells were cultured for 14 days in 0.5 mM butyrate or 9 days in 0.5 μM TSA. These concentrations allowed for the prolonged exposure of WI-38 cells to the HDAC inhibitors with minimal cytotoxicity. As shown in Figure 2, young WI-38 cells cultured in the presence of either HDAC inhibitor acquired the perinuclear staining for SA-β-gal activity normally associated with senescent cells. Untreated proliferating WI-38 cells propagated in parallel had no SA-β-gal activity (Figure 2). This data further supports the findings that HDAC inhibition induces a senescent-like phenotype in proliferating fibroblasts [26,27].

### Proteasome activity is reduced in senescent WI-38 cells

**Figure 3:**
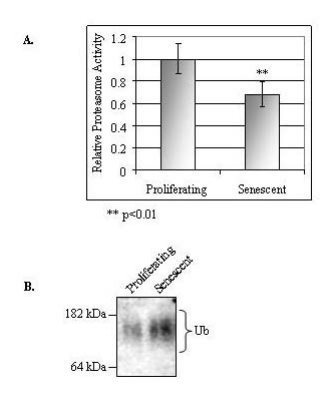
**Proteasome activity is reduced in senescent WI-38 cells**. **(A) **Cell lysates were prepared from proliferating or senescent WI-38 cells. Protein concentrations were normalized and proteasome activity was determined using a synthetic fluorogenic substrate (± standard error; n = 4). The reduction in proteasome activity was found to be significant in the older cells (**, p < 0.01). **(B) **Polyubiquitinated proteins found in the cytosolic extracts of proliferating and senescent WI-38 cells. Sample concentrations were normalized and the level of polyubiquitin-protein conjugates was determined by immunoblot using an antibody specific for ubiquitin.

Declines in proteasome function during senescence and aging have been observed in cultured cells and in tissues from a variety of organisms [8-10,14,15,17]. Our aim was to verify and characterize the changes in proteasome activity following senescence in the human fibroblast WI-38 cell line. Cytosolic extracts prepared from proliferating and senescent WI-38 cells were tested for proteasome activity using the synthetic substrate Suc-LLVY-AMC [30]. As shown in Figure 3A, proteasome activity was significantly lower (reduced by ~30%) in the older WI-38 cells. Since a decrease in proteasome activity may also cause a general increase in the presence of polyubiqitinated proteins, cytsolic extracts from proliferating and senescent WI-38 cells were analyzed by immunoblotting for polyubiquitinated proteins. As indicated in Figure 3B, the accumulation of high molecular weight ubiquitin-conjugated proteins was accentuated in the senescent WI-38 cells, which is consistent with a drop in proteasome activity.

**Figure 4:**
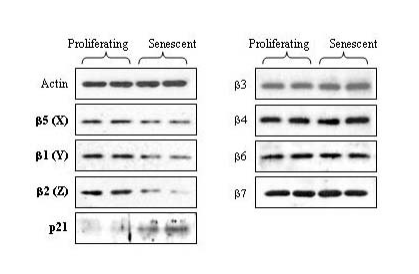
**Expression levels of the β-type proteasome subunits in proliferating and senescent WI-38 cells**. Cytosolic extracts were prepared from proliferating and senescent WI-38 cells. Samples were analyzed by immunoblot using antibodies specific for each of the β-type proteasome subunits. Actin served as a loading control. Levels of p21^WAF1 ^(p21) were also determined to serve as an inducible marker for replicative senescence. Results from duplicate cultures are shown.

**Figure 5:**
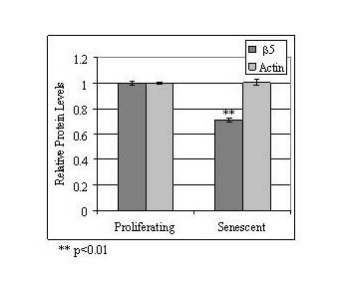
**Quantified levels of the β5 proteasome subunit and actin protein in proliferating and senescent WI-38 cells**. Cytosolic extracts were prepared and equivalent protein levels were analyzed for β5 subunit expression by immunoblotting. Actin levels served as a loading control. The graphical display indicates the relative intensities of β5 and actin levels determined by optical densitometry from corresponding immunoblots (± standard error; n = 3). The decline in β5 levels was found to be significant in senescent cells (**, p < 0.01).

Previous reports have documented that certain proteasome subunits are down-regulated in senescent WI-38 cells [14,15]. However, these analyses were limited to only a select subset of proteasome subunits. We therefore analyzed the expression of each constitutive β-type subunit to further characterize differences in proteasome subunit expression between proliferating and senescent cells (Figure 4). As shown in Figure 4, senescent WI-38 cells expressed lower levels of the three catalytic proteasome subunits: β5 (X), β1 (Y), and β2 (Z). However, the expression levels of the other β-type subunits did not change in the older cells. Figure 4 also shows the increased expression of p21^WAF1 ^in senescent WI-38 cells [20]. The protein expression levels of the β5 subunit were additionally quantified by optical densitometry from immunoblots (Figure 5). The β5 protein levels were reduced by ~30% in senescent WI-38 cells, which corresponds to the ~30% decline in proteasome activity (as shown in Figure 3A).

### Senescent WI-38 cells are resistant to HDAC inhibitors

**Figure 6:**
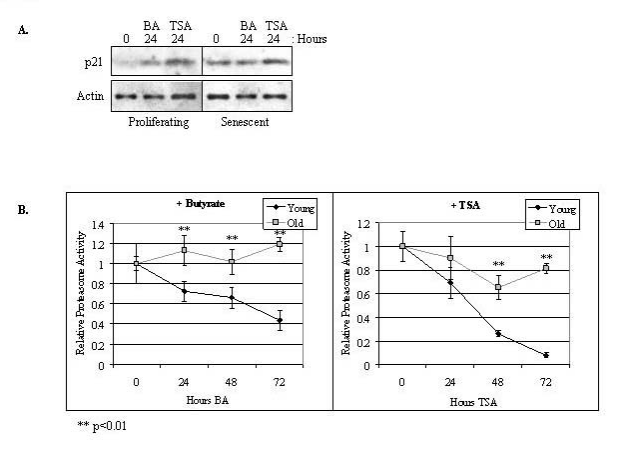
**Senescent WI-38 cells are resistant to the effects of HDAC inhibition**. **(A) **Butyrate and TSA did not further enhance the expression of cell cycle inhibitor protein p21^WAF1 ^in senescent WI-38 cells. Proliferating and senescent WI-38 cells were treated with butyrate (BA) or TSA for 24 hours, as indicated. Cytosolic extracts were prepared and equivalent protein levels were analyzed by immunoblot using an antibody specific for p21^WAF1^. Actin served as a loading control and was determined by immunoblotting for the actin protein. Control samples (0 hours) consisted of cytosolic extracts prepared from untreated cells. **(B) **The effect of HDAC inhibitors on proteasome activity in proliferating and senescent WI-38 cells. Cytoslic extracts were prepared from proliferating (Young;◆) and senescent (Old;) WI-38 cells treated with butyrate (+ Butyrate) or TSA (+ TSA) for 0, 24, 48 and 72 hours, as indicated. Equivalent protein concentrations were determined and analyzed for proteasome activity using a synthetic fluorogenic substrate (± standard error; n = 4; values of non-treated samples set to 1). Senescent cells were found to be significantly less responsive to proteasome inhibition by butyrate and TSA.

The expression of p21^WAF1 ^is regulated by aceytlation and readily activated by HDAC inhibitors [31,32]. We therefore determined the effect of HDAC inhibitors on p21^WAF1 ^expression in proliferating and senescent WI-38 cells. As shown in Figure 6A, treatment with HDAC inhibitors butyrate or TSA induced p21^WAF1 ^expression in proliferating WI-38 cells. However, in senescent cells the endogenous levels of p21^WAF1 ^were high and not further enhanced by either HDAC inhibitor (Figure 6A). These data suggest that p21^WAF1^activation in senescent cells may result from a reduction in cellular HDAC activity.

HDAC inhibitors have also been reported to suppress proteasome activity and subunit expression in several transformed cell lines [33-35]. We hypothesized that HDAC inhibitors may suppress proteasome activity in proliferating WI-38 cells, as well. Cytosolic extracts were prepared from young WI-38 cells treated with butyrate or TSA for 0, 24, 48, and 72 hours. The synthetic substrate Suc-LLVY-AMC was then utilized to measure proteasome activity in each sample. As shown in Figure 6B, proteasome activity decreased in young WI-38 cells treated with either butyrate or TSA. To determine if senescent WI-38 cells were also sensitive to HDAC inhibitor-induced proteasome suppression, the proteasome activity of senescent WI-38 cells was analyzed following butyrate or TSA treatment. Although proteasome activity was lower in senescent WI-38 cells (as shown in Figure 3A), it was significantly less sensitive to the inhibitory effects of the HDAC inhibitors (Figure 6B). This data suggests that replicative senescence and HDAC inhibitor-induced senescence impacts proteasome activity through a common pathway.

### Reduced expression of the β5 proteasome subunit in proliferating WI-38 cells by HDAC inhibitors

**Figure 7:**
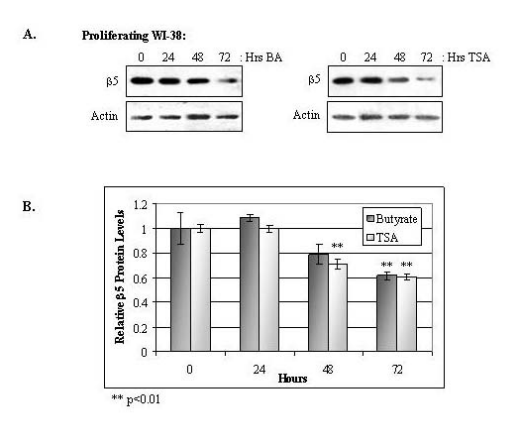
**HDAC inhibitors down-regulate β5 proteasome subunit expression in proliferating WI-38 cells**. **(A) **Proliferating WI-38 cells were treated with butyrate (BA) or TSA for 0, 24, 48, or 72 hours, as indicated. Cytosolic extracts were prepared and equivalent protein levels (determined by a Bradford assay) were analyzed by immunoblotting using an antibody specific for the β5 proteasome subunit. Actin served as a loading control. **(B) **Immunoblots from Figure 7A were quantified by optical densitometry. The graphical display shows the relative levels of the β5 subunit (normalized to actin) from the corresponding immunoblots (± standard error; n = 3). The decline in β5 levels was found to be significant at the indicated time points (**, p < 0.01).

We determined whether the HDAC inhibitors butyrate and TSA could suppress the expression of the catalytic β5 subunit of the proteasome in proliferating WI-38 cells. The immunoblots in Figure 7A indicate that β5 expression levels decreased in these cells following butyrate or TSA treatments. The expression levels of the β5 subunit were additionally quantified by optical densitometry from immunoblots (Figure 7B). This data indicates that reduced proteasome activity following HDAC inhibition (Figure 6B) may be due in part to reduced proteasome subunit expression.

### HDAC1 is down-regulated in senescent WI-38 cells

**Figure 8:**
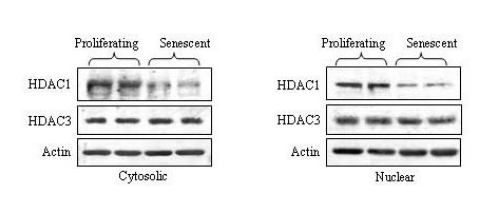
**HDAC1 and HDAC3 levels in proliferating and senescent WI-38 cells**. Cytosolic and nuclear extracts were prepared from proliferating and senescent WI-38 cells. HDAC1 and HDAC3 levels were determined in both fractions by immunoblotting. Actin served as a loading control. The results from duplicate cultures are shown.

The class I histone deacetylase protein HDAC1 is a component of the corepressor complex involved in suppressing the transcription of p21^WAF1 ^and other cell cycle inhibitory genes [22]. We therefore determined if HDAC1 expression was altered upon replicative senescence. As shown in Figure 8, HDAC1 levels decreased in senescent WI-38 cells. (It should be noted that HDAC1 is predominantly a nuclear protein, but diffuses into the cyoplasmic fraction during protein extraction.) The levels of another class I histone deacetylase, HDAC3, was found to be equivalent in pre- and post-senescent cells (Figure 8). The drop in HDAC1 expression may contribute to the induction of p21^WAF1 ^in senescent WI-38 cells (as shown in Figure 4; panel p21). Likewise, the decline in HDAC1 may contribute to the appearance of other senescent phenotypes, such as a drop in proteasome activity.

## Discussion

**Figure 9:**
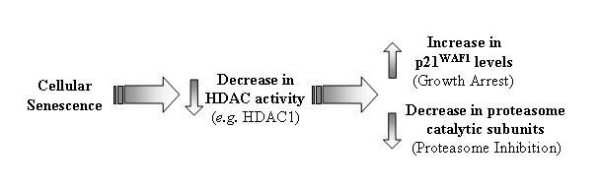
**Model: the age-related decline in HDAC levels contributes to replicative senescence**. As WI-38 cells enter senescence, cellular HDAC activity is envisioned to decrease to alter gene expression. The reduction in HDAC activity induces the expression of p21^WAF1 ^to assist in senescence-associated growth arrest. It is also envisioned that the decline in HDAC activity contributes to the senescence-associated drop in proteasome activity.

Replicative senescence marks the end of the proliferative life span of normal cells. This is accompanied by distinct alterations in the pattern of gene expression. It has been suggested that changes in gene expression during senescence and aging may result in part from alterations in protein acetylation [36-38]. Figure 9 illustrates a potential mechanism by which HDACs (*e.g. *HDAC1) contribute to the senescence phenotype. As WI-38 cells senesce, HDAC activity decreases to facilitate changes in gene expression. Reductions in HDAC levels, in association with increased transcriptional activity of p53 in senescent cells, contributes to the induction of p21^WAF1 ^expression and subsequent growth arrest [39,40]. This model also envisions HDACs contributing to the age-related decline in proteasome activity, since HDAC inhibitors can reduce proteasome expression and activity [33-35,41,42].

The identification of HDACs as a component in replicative senescence, and hence growth arrest, is interesting because data has shown that HDACs can promote tumor growth and stem cell proliferation. For example, it has been reported that HDAC1 overexpression occurs in 68% of primary human gastric cancer, and contributes to colony formation and proliferation of prostate and breast cancer cells [43-45]. Some transformed cell types may exaggerate the expression of HDACs to circumvent replicative senescence. In this regard, cancer cells are similar to stem cells, where HDAC1 is required for full cellular growth potential [46]. This further supports the idea that replicative senescence, and the associated decline in HDAC1 expression, has a tumor suppressing role [4,47].

It is not entirely clear how HDACs are regulating proteasome subunit expression. In yeast, a common mode of transcriptional regulation of the proteasomal subunits has already been identified [48-50]. Nearly all the yeast subunit homologs have been found to possess proteasome-associated control elements within their promoters. The transcription factor RPN4 has been identified as the component within yeast involved in binding these elements to modulate gene transcription [48]. Remarkably, no homolog of RPN4 has been identified in humans. However, it is still possible that another common transcriptional mechanism is shared amongst the catalytic subunits in human cells. The activity of these putative regulatory proteins may be regulated by acetylation, such that an increased level of acetylation reduces proteasome subunit expression.

Our analysis of HDAC1 and HDAC3 indicates that replicative senescence is not accompanied by a global decline in HDAC expression. Rather, it appears to occur through the down-regulation of HDAC1, and potentially other HDACs. Other groups have also reported a senescence-specific form of the HDAC2 protein [38]. In addition, the NAD^+^-dependent Sir2 histone deacetylase has been identified to contribute to the replicative life-span in yeast, thus suggesting that the mammalian Sir2-related class III HDACs may contribute to senescence in normal human cell types, as well [51,52]. It should be noted that it is not clear if the decline in HDAC1 is a cause or a consequence of replicative senescence. However, it seems reasonable to hypothesize that age-related modulations in HDAC levels could be a contributing factor in senescence. Further analysis of individual HDAC proteins may identify their individual functions within the senescence machinery. Anti-aging and anti-cancer strategies may be aimed at increasing or decreasing the activity of specific HDAC proteins.

## Conclusion

Our findings indicate that cellular HDAC activity regulates numerous phenotypic changes associated with cellular senescence. Reduced cellular HDAC expression and activity, in association with other events, may be important for mediating the transition to a senescent phenotype.

## Methods

### Cell culture and treatments

The WI-38 human lung fibroblast cell line was purchased from American Type Culture Collection (Manassas, VA). Cells were propagated in minimal essential media containing 2 mM L-glutamine and Earle's salts (E-MEM) supplemented with 10% fetal bovine serum, 0.1 mM non-essential amino acids, 1 mM Sodium Pyruvate, streptomycin (50 mg/ml), and penicillin (50 U/ml). All medium components were purchased from Invitrogen Life Technologies (Carlsbad, CA). WI-38 cells entered senescence at about 50 CPD (Cumulative Population Doublings). Early-passage WI-38 cells (CPD < 30) are referred to as young or pre-senescent cells and displayed high proliferative potential. Late-passage WI-38 cells (CPD > 50) are classified as old or post-senescent cells and exhibited very low proliferative potential. Sodium butyrate (Sigma-Aldrich, St. Louis, MO) was used at the final concentration of 4 mM (unless stated otherwise). TSA (Calbiochem, San Diego, CA) was used at a 2 μM concentration (unless stated otherwise). Cells treated with TSA were given fresh media supplemented with new TSA every 24 hours.

### Immunoblotting

Cytosolic extracts were prepared as described in Inan et al. [53]. For immunoblotting studies, 25 μg of cytoplasmic protein (quantified by the Bio-Rad protein assay) was denatured under reducing conditions, separated on 10% sodium dodecyl sulfate (SDS) polyacrylamide gels, and transferred to nitrocellulose by voltage gradient transfer. The resulting blots were blocked with 5% nonfat dry milk. Specific proteins were detected with appropriate antibodies using enhanced chemiluminescence detection (Santa Cruz Biotechnology, Santa Cruz, CA). Immunoblotting antibodies used were: subunit β1 PW8140, subunit β2 PW8145, subunit β3 PW8130, subunit β4 PW8890, subunit β5 PW8895, subunit β6 PW9000, and subunit β7 PW8135, (Affiniti Research Products Ltd., Mamhead, Exeter, UK); Ubiquitin P1A6, (Santa Cruz Biotechnology, Santa Cruz, CA); p21 C-19, (Santa Cruz Biotechnology, Santa Cruz, CA); and Actin I-19, (Santa Cruz Biotechnology, Santa Cruz, CA). The antibodies specific for ubiquitin and p21^WAF1 ^were diluted 1:500 for immunoblotting. All other antibodies were employed at a 1:1000 dilution. For optical densitometry, immunoblot images were scanned on a UMAX Astra 1220P scanner and analyzed with NIH Image version 1.62. Statistical significance was determined by a paired Student's *t*-test.

### Proteasome activity assay

Proteasome activity was quantified by using a fluorogenic proteasome-specific substrate. The assay is based on the detection of the fluorophore AMC (7-amino-4-methylcoumarin) after cleavage from the synthetic proteasome substrate Suc-LLVY-AMC (Calbiochem, San Diego, CA). Cytosolic extract (5 μg of total protein in 5 μl) was incubated in a 100 μl reaction containing 20 mM Tris-HCL (pH 7.8), 0.5 mM EDTA, 0.035% SDS, and 70 μM Suc-LLVY-AMC for 10 minutes at room temperature. The change in fluorescence (substrate consumption) was measured over an interval of 40 minutes using a microtiter plate fluorometer (excitation, 360 nm; emission, 460 nm). Proteasome-independent activity was determined by performing the assay in the presence of proteasome inhibitor MG-132 (final concentration 60 μM) (Calbiochem, San Diego, CA). Proteasome activity values were derived by subtracting the fluorescence obtained in the presence of this inhibitor from the values obtained in its absence. The values shown represent the ratio in proteasome activity from each sample compared to the activity in young WI-38 cell extracts. Assays were performed in quadruplicate, and statistical significance was determined by a paired Student's *t*-test.

### Senescence-associated β-galactosidase staining

Staining for β-galactosidase activity in WI-38 cells was performed as previously described [29]. WI-38 cells were washed with PBS, fixed in 0.2% glutaraldehyde/2% formaldehyde for 10 minutes at room temperature, and washed again with PBS. Cells were then stained at 37°C (in the absence of CO_2_) with fresh senescence-associated β-gal (SA-β-gal) staining solution (150 mM NaCl, 2 mM MgCl_2_, 5 mM potassium ferricyanide, 5 mM potassium ferrocyanide, and 40 mM citric acid/sodium phosphate, pH 6.0) containing 1 mg/ml 5-bromo-4-chloro-3-indolyl-β-D-galactoside (X-gal). Once staining was maximal (12–16 hrs), cells were washed with PBS and overlaid in 70% glycerol. Images were taken at 100 × magnification as viewed by phase contrast.

## Abbreviations

HDAC, histone deacetylase; BA, butyrate; TSA, trichostatin A; SA-β-gal, senescence-associated-β-galactosidase; E-MEM, minimal essential media with Earle's salts; Suc-LLVY-AMC, *N*-succinyl-Leu-Leu-Val-Tyr-7-amino-4-methylcoumarin.

## Authors' contributions

RFP performed all the experiments, designed the study, and wrote the manuscript. EJN helped capture all microscopic images and discussed these results. CG was the principal investigator who gave advice in designing the study and edited the manuscript.
